# Platelet-Derived Growth Factor Receptor Beta: A Novel Urinary Biomarker for Recurrence of Non-Muscle-Invasive Bladder Cancer

**DOI:** 10.1371/journal.pone.0096671

**Published:** 2014-05-06

**Authors:** Jiayu Feng, Weifeng He, Yajun Song, Ying Wang, Richard J. Simpson, Xiaorong Zhang, Gaoxing Luo, Jun Wu, Chibing Huang

**Affiliations:** 1 Department of Urology, Xinqiao Hospital, The Third Military Medical University, Chongqing, China; 2 Chongqing Key Laboratory for Disease Proteomics; State Key Laboratory of Trauma, Burns and Combined Injury, Institute of Burn Research, Southwest Hospital, Third Military Medical University, Chongqing, China; 3 Department of Biochemistry, La Trobe Institute for Molecular Science, La Trobe University, Bundoora, Victoria, Australia; Louisiana State University Health Sciences center, United States of America

## Abstract

Non-muscle-invasive bladder cancer (NMIBC) is one of the most common malignant tumors in the urological system with a high risk of recurrence, and effective non-invasive biomarkers for NMIBC relapse are still needed. The human urinary proteome can reflect the status of the microenvironment of the urinary system and is an ideal source for clinical diagnosis of urinary system diseases. Our previous work used proteomics to identify 1643 high-confidence urinary proteins in the urine from a healthy population. Here, we used bioinformatics to construct a cancer-associated protein-protein interaction (PPI) network comprising 16 high-abundance urinary proteins based on the urinary proteome database. As a result, platelet-derived growth factor receptor beta (PDGFRB) was selected for further validation as a candidate biomarker for NMIBC diagnosis and prognosis. Although the levels of urinary PDGFRB showed no significant difference between patients pre- and post-surgery (n = 185, P>0.05), over 3 years of follow-up, urinary PDGFRB was shown to be significantly higher in relapsed patients (n = 68) than in relapse-free patients (n = 117, P<0.001). The levels of urinary PDGFRB were significantly correlated with the risk of 3-year recurrence of NMIBC, and these levels improved the accuracy of a NMIBC recurrence risk prediction model that included age, tumor size, and tumor number (area under the curve, 0.862; 95% CI, 0.809 to 0.914) compared to PDGFR alone. Therefore, we surmise that urinary PDGFRB could serve as a non-invasive biomarker for predicting NMIBC recurrence.

## Introduction

Bladder cancer is the seventh most prevalent cancer worldwide, and approximately 80% of cases are non-muscle-invasive bladder cancer (NMIBC), while the remaining 20% are muscle-invasive bladder cancer (MIBC)[1]–[3]. Of all newly diagnosed cases of transitional cell carcinomas, approximately 80% present as non-muscle-invasive tumors (Ta–T1)[3]. For NMIBC, 50–70% of patients will develop disease recurrence within two years of their initial diagnosis[4]. Current clinical and conventional histopathological parameters such as tumor stage, grade and size of tumors, are well studied in terms of providing prognostic information regarding progression to muscle invasion and recurrence. However, none of these factors have proven to be sufficient to predict the diverse behavior of NMIBC. Thus, biomarkers for NMIBC recurrence, which are non-invasive, are urgently required for clinical treatment.

Human urine is one of the major body fluids and is an ideal source for clinical diagnosis, especially for urinary system diseases, as urine can be obtained non-invasively in large quantities[5]. Urinary proteins should be regarded as potential sources of biomarkers for several diseases. Over the past few years, great technological advances have occurred in proteomics, and a large number of proteins in the urinary proteome of healthy people have been identified[6]–[10]. However, the application of such a urinary protein databases to facilitate identification of biomarkers for diseases has experienced limited progress. Thus far, protein–protein interaction (PPI) data has been widely used for the identification of biomarkers with the assumption that interaction proteins significantly reflect disease status because proteins do not function in isolation, but rather interact with one another. Additionally, they can provide hypotheses such as signaling pathways and other mechanisms that impact the disease outcome. Construction of a PPI network could highlight the disease-associated proteins that have biological function. In this study, we sought to screen potential biomarkers for NMIBC diagnosis and prognosis by constructing a cancer-associated PPI network based on the healthy human urinary proteome, and we revealed that PDGFRB could serve as a prognostic biomarker for NMIBC recurrence.

## Materials and Methods

### Ethics statement

The study was approved by the Ethics Committee of the Xinqiao Hospital, Chongqing and adhered to the tenets of the Declaration of Helsinki. In addition, written informed consent was obtained by the patients in this study.

### High abundance urinary proteins dataset

Our previous study identified 1641 high-confidence urinary proteins in the healthy population [11]. The definition of a high-abundance protein was arbitrarily set as one with >4 unique peptides and a spectra number in the database of >10. A total of 592 high-abundance urinary proteins were obtained for further bioinformatics analysis (File S2).

### Extracellular and plasma membrane proteins were obtained by GO analysis

We used the BiNGO plug-in to find statistically over-represented GO categories in the biologic data as a tool for enrichment analysis of the high-abundance urinary protein dataset. For enrichment analysis, we needed a test dataset (which contained 592 high-abundance urinary proteins) and a reference set of GO annotations for the complete human proteome. The analysis was performed using a “hyper-geometric test”, and all GO terms that were significant with P<0.0001 (after correcting for multiple testing using the Benjamini and Hochberg false discovery rate corrections) were selected as over-represented. Then, the proteins that were annotated as “extracellular” and “plasma membrane” were selected for PPI network construction.

### PPI network construction

Protein-protein interactions were predicted using the Search Tool for the Retrieval of Interacting Genes/Proteins (STRING) database v9.0 (http://www.string-db.org/). Proteins were linked based on the following six criteria; neighborhood, gene fusion, co-occurrence, co-expression, experimental evidence and existing databases[12].

### Cancer-associated proteins in the PPI network were analyzed by KEGG pathway enrichment

We used ClueGO, an easy-to-use Cytoscape plug-in[13], to integrate Gene Ontology (GO) terms as well as the Kyoto encyclopedia of genes and genomes (KEGG)/BioCarta pathways to create a functionally organized GO/pathway term network for the protein network in urine. The enrichment tests for terms and groups were two-sided (Enrichment/Depletion) tests based on the hyper-geometric distribution, and all cancer-associated terms that were significant with P<0.05 (after correcting for multiple testing using the Benjamini and Hochberg false discovery rate corrections) were selected for further analysis. The Kappa Score Threshold was set to 0.5. ClueGO visualizes the selected terms in a functionally grouped annotation network that reflects the relationships between the terms based on the similarity of their associated proteins.

### Study participants

A total of 185 histologically demonstrated NMIBC cases were selected from January 2007 to January 2010. The criteria for study enrollment were as follows: histopathological diagnosis of transitional cell carcinoma of the bladder that was newly diagnosed and untreated, no history of other tumors, and the ability to conduct follow-up. The patients included 160 men and 25 women from 32 to 83 years (mean age, 62.1 years). All patients underwent transurethral resection of bladder tumor (TURBT). All patients with NMIBC received intravesical mitomycin C (MMC) or pirarubicin (THP) instillations once weekly for the first 8 weeks and then monthly up to 1 year. We defined recurrence as the recurrence of primary NMIBC with an equal or lower pathologic stage. Other clinical and pathological features of the enrolled patients are summarized in Table 1. The grade and stage of the patients were defined according to the WHO 1973 criteria for grade and the 2002 TNM classification system.

**Table 1:** Patient demographics and clinical parameters.

	Relapse	No relapse
Number of patients	68	117
Age (≤ 60/>60)	28/40	52/65
Gender (male/female)	57/11	103/14
Pathological stage Ta/T1	25/43	49/68
Grade (I/II)	24/44	43/74
Unifocal/Multifocal	30/38	75/42
Tumor size (≤ 3 cm/>3 cm)	43/25	96/21
Time to relapse (months)	11.2±4.3	N/A

### Urine sample preparation

Morning urine samples were collected daily over the 3 days before primary surgery and over the 3 days after the surgery. The urine samples were stored at −80°C until all samples were collected. Ten milliliters of urine was taken from each sample before surgery and after surgery and pooled together. The urine samples were centrifuged at 10,000 × g for 30 min at 4°C to remove any cellular debris, and the supernatant was concentrated and desalted by using a membrane with a cutoff of 10 kDa. The protein amount in the urine concentrates was measured using a Coomassie Protein Assay Kit (Pierce, Rockford, IL, USA). Soon after, the urine concentrates were frozen at −80°C.

### PDGFRB expression analysis on NMIBC tissues

The NMIBC tissues from relapse and relapse-free patients were selected for immunohistochemical validation. All tissues were fixed with 10% formaldehyde and paraffinembedded. The tissues were cut at 4µm in thickness. The antibody for PDGFRB was purchased from Abcam. A two-step immunohistochemical technique was used. Briefly, the sections were dewaxed and hydrated, and then boiled in 10 mM citrate buffer, pH 6.0 for 10 min. Blocking was performed with nonspecific binding with 5% (v/v) bovine serum albumin (BSA) for 10 min. The sections were incubated with PDGFRB Ab (1∶200 working dilution) at 4°C for 12 h in a moist chamber. After washing with 0.02M phosphate buffer saline (PBS) pH 7.4 three times, the sections were incubated with secondary antibody conjugated with HRP-labeled polymer (Abcam) at 37 °C for 30 min. The sections were then incubated with liquid DAB substrate-chromogen for 10 min at room temperature, rinsed in distilled water, and counterstained with hematoxylin.

### Validation by Western Blot

Approximately 20 µg urinary protein was separated by 12% SDS-PAGE, then transferred to a PVDF membrane (Millipore, USA), and probed with polyclonal rabbit anti-human PDGFRB proteins antibody (1∶2000, Abcam, USA), respectively. The blots were labeled with horseradish peroxidase-conjugated secondary antibodies (1∶10000), and visualized with enhanced chemiluminescence (ECL) detection system (Pierce Biotech Inc., Rockford, IL).

### ELISA

The protein amount in the urine concentrates was measured using a Coomassie Protein Assay Kit (Pierce, Rockford, IL, USA). Human PDGFRB protein was quantified with ELISA kits from Uscn Life Science Inc. (Wuhan, China) according the manufacturer's instructions.

### Statistical analysis

An unpaired t-test and one-way ANOVA were used to analyze the correlation between the groups. A stepwise model selection process was used to arrive at a parsimonious model. The logistic regression modeling was employed to describe the relationship between levels of urinary PDGFRB and the risk of breast cancer recurrence and other clinical observations. Receiver operating characteristic (ROC) curves were plotted to evaluate the sensitivity and specificity of the biomarker measurements in predicting the recurrence of NMIBC. Two-tailed P values less than 0.05 were considered significant. Continuous variables are expressed as the mean ± the standard deviation unless otherwise indicated.

## Results

### Construction of a cancer-associated urinary PPI network based on our previous urinary proteome database

We previously reported that 1641 high-confidence urinary proteins were identified in a healthy human population by four fractionation techniques (in-gel, 2DLC, OFFGEL and mRP) coupled with HPLC-CHIP-MS/MS[11]. Herein, we sought to find non-invasive prognostic biomarker candidates for NMIBC recurrence through bioinformatics analysis of the healthy population urinary proteome. To find urinary protein biomarkers that are suitable for routine clinical examination methods, we first screened for high-abundance urinary proteins in the urinary proteome database, which by our definition had >4 unique peptides and had a spectra number >10. A total of 592 high-abundance urinary proteins were obtained from the urinary proteome database for further analysis (File S2).

**Figure 1:**
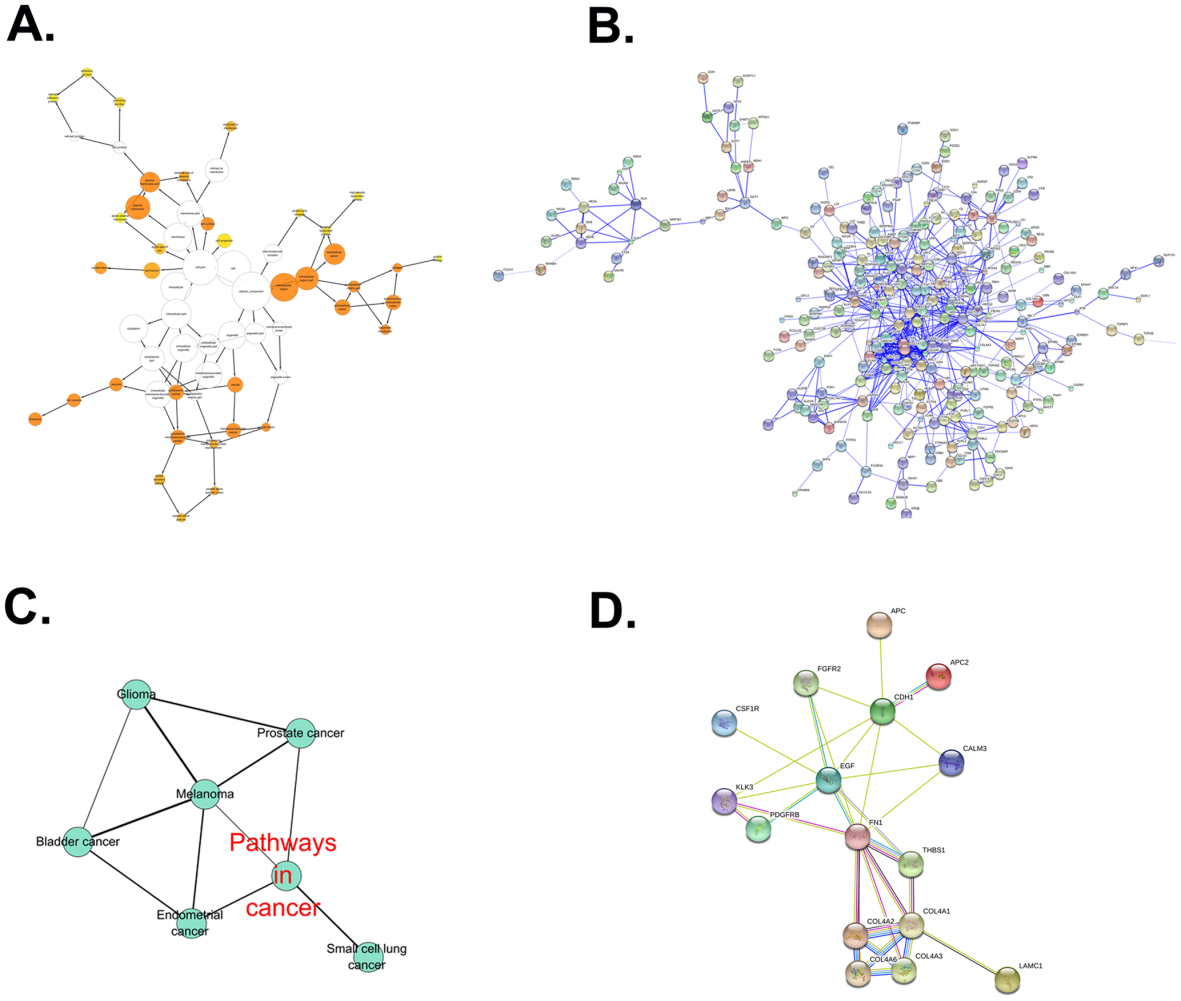
A cancer-associated PPI network in urine was constructed using a bioinformatics approach. (A) A total of 592 high-abundance urinary proteins in our urinary proteome database were selected according to the criteria of having >4 unique peptides and a spectra number >10. Those proteins were analyzed using the BinGO plug-in in Cytoscape software, and 373 proteins were annotated as being “extracellular region” and “plasma membrane.” A map of the cellular component is shown. (B). The protein-protein interaction networks of the 373 proteins were constructed by STRING. A PPI network, which was composed of 312 nodes and 1779 interactions with removal of single nodes, was obtained. (C) The PPI network was analyzed by Cytoscape software with the ClueGO+Cluepedia plug-in. Six cancer-associated enriched KEGG terms were obtained and shown. (D) A cancer-associated urinary PPI network comprised of 15 urinary proteins that were associated with the 6 cancer-associated terms were constructed by STRING.

To avoid contamination with cellular debris, the proteins that were annotated as “extracellular” and “plasma membrane” among the total urinary proteins were selected by the BiNGO analysis. In total, 544 proteins were linked to at least one annotation term within the GO cellular component. In total, 37 terms exhibited significance (P<0.0001) as being over-represented and under-represented terms compared with the entire list of International Protein Index (IPI) entries. More detailed information regarding the GO analysis is available as File S2. As shown in Figure 1A, in the cellular component category, GO terms related to extracellular proteins such as the extracellular region (269 proteins found), the extracellular space (140), the extracellular matrix (68), and the plasma membrane (197) were over-represented, as was expected. A total of 373 urinary proteins that were annotated as “extracellular region” and “plasma membrane” were obtained for further PPI network construction (File S2). Because the PPI network is significant to reflect the disease status compared with single proteins, the 373 proteins were used to construct a PPI network through STRING analysis. Single nodes and small components of the PPI network that were initially assembled were removed, and only the largest component was saved as a new PPI network, which was composed of 312 nodes and 1779 interactions (Figure 1B). The PPI network information is described in detail as File S2. To further investigate cancer-associated proteins in this sub-network, the 312 encoded proteins were analyzed using the Cytoscape plug-in ClueGO+CluePedia. An annotation network based on KEGG pathways was created as a group of functionally organized GO/pathway terms network (Figure S1 in File S1). The term network information is described in detail as File S2. Among these term networks, a functionally grouped cancer-associated network is shown in figure 1C. It comprises 6 specific significantly overrepresented terms such as “pathways in cancer”, “endometrial cancer”, “prostate cancer”, “glioma”, “melanoma”, “bladder cancer” and “small cell lung cancer”. The information regarding the cancer terms in the associated KEGG network is described in detail in Table 2. Furthermore, a total of 15 proteins, which were associated with all 6 cancer terms (Table 3), were used to construct a cancer-associated urinary PPI network (Figure 1D). It was further observed that EGF and CDH1 in the PPI network were annotated as being bladder cancer-associated proteins, and both receptor FGFR2 and PDGFRB could interact with EGF (Table 3). Because PDGFRB/EGFR heterodimers were reported to express on bladder cancer cells[14], and the EGF/EGFR pathway played an important role in bladder cancer development[15], [16], PDGFRB was selected for further validation as a candidate biomarker of bladder cancer.

**Table 2:** Cancer-associated KEGG/GO terms.

GOTerm	Nr. Genes	Term P value	Group P value	Associated genes found
Pathways in cancer	15	2.9 E-2	2.0 E-11	[APC, APC2, CDH1, COL4A1, COL4A2, COL4A3, COL4A6, SF1R, CTNNA3, EGF, FGFR2, FN1, KLK3, LAMC1, PDGFRB]
Endometrial cancer	5	1.2 E-2	2.0 E-11	[APC, APC2, CDH1, CTNNA3, EGF]
Glioma	3	2.5 E-1	2.0 E-11	[CALM3, EGF, PDGFRB]
Prostate cancer	4	2.1 E-1	2.0 E-11	[EGF, FGFR2, KLK3, PDGFRB]
Melanoma	3	2.9 E-1	2.0 E-11	[CDH1, EGF, PDGFRB]
Bladder cancer	3	8.1 E-2	2.0 E-11	[CDH1, EGF]
Small cell lung cancer	6	2.7 E-2	2.0 E-11	[COL4A1, COL4A2, COL4A3, COL4A6, FN1, LAMC1]

**Table 3:** Cancer-associated urinary PPI network.

#node1	node2	cooccurence	homology	coexpression	experimental	knowledge	textmining	combined_score
COL4A1	THBS1	0	0	0.104	0.62	0	0.29	0.724
COL4A3	COL4A1	0.525	0.936	0	0	0.9	0.66	0.907
FGFR2	EGF	0	0	0	0	0.8	0.528	0.899
COL4A1	COL4A2	0.525	0.927	0.776	0.999	0.9	0.87	0.999
COL4A6	COL4A2	0.525	0.933	0	0	0.9	0.675	0.907
FN1	KLK3	0	0	0	0.621	0	0.374	0.746
CSF1R	EGF	0	0	0	0	0	0.43	0.43
EGF	THBS1	0	0.442	0	0	0	0.885	0.521
FGFR2	FN1	0	0	0	0	0	0.43	0.43
COL4A1	COL4A6	0.525	0.925	0	0	0.9	0.672	0.908
COL4A3	COL4A6	0.525	0.92	0	0	0.9	0.791	0.909
CDH1	APC	0	0	0	0	0	0.67	0.669
COL4A3	COL4A2	0.525	0.917	0	0	0.9	0.718	0.909
COL4A1	LAMC1	0	0	0.215	0	0	0.34	0.447
CDH1	APC2	0	0	0	0.769	0.72	0.176	0.939
COL4A3	FN1	0	0	0	0.62	0	0.341	0.732
EGF	CDH1	0	0	0	0	0	0.927	0.927
COL4A1	FN1	0	0	0.18	0.62	0	0.379	0.779
EGF	PDGFRB	0	0	0	0	0.8	0.412	0.874
FN1	THBS1	0	0.406	0.13	0.846	0.72	0.917	0.98
COL4A2	FN1	0	0	0.163	0.62	0	0.275	0.737
KLK3	CDH1	0	0	0	0	0	0.469	0.469
FN1	EGF	0	0	0	0	0.9	0.946	0.994
FN1	CDH1	0	0	0	0	0	0.752	0.752
KLK3	EGF	0	0	0	0	0	0.463	0.462
FGFR2	CDH1	0	0	0	0	0	0.467	0.467
KLK3	PDGFRB	0	0	0	0.621	0	0.068	0.623
COL4A6	FN1	0	0	0	0.62	0	0.148	0.654

### There were no significant differences of urinary PDGFRB between patients pre- and post-surgery

**Figure 2:**
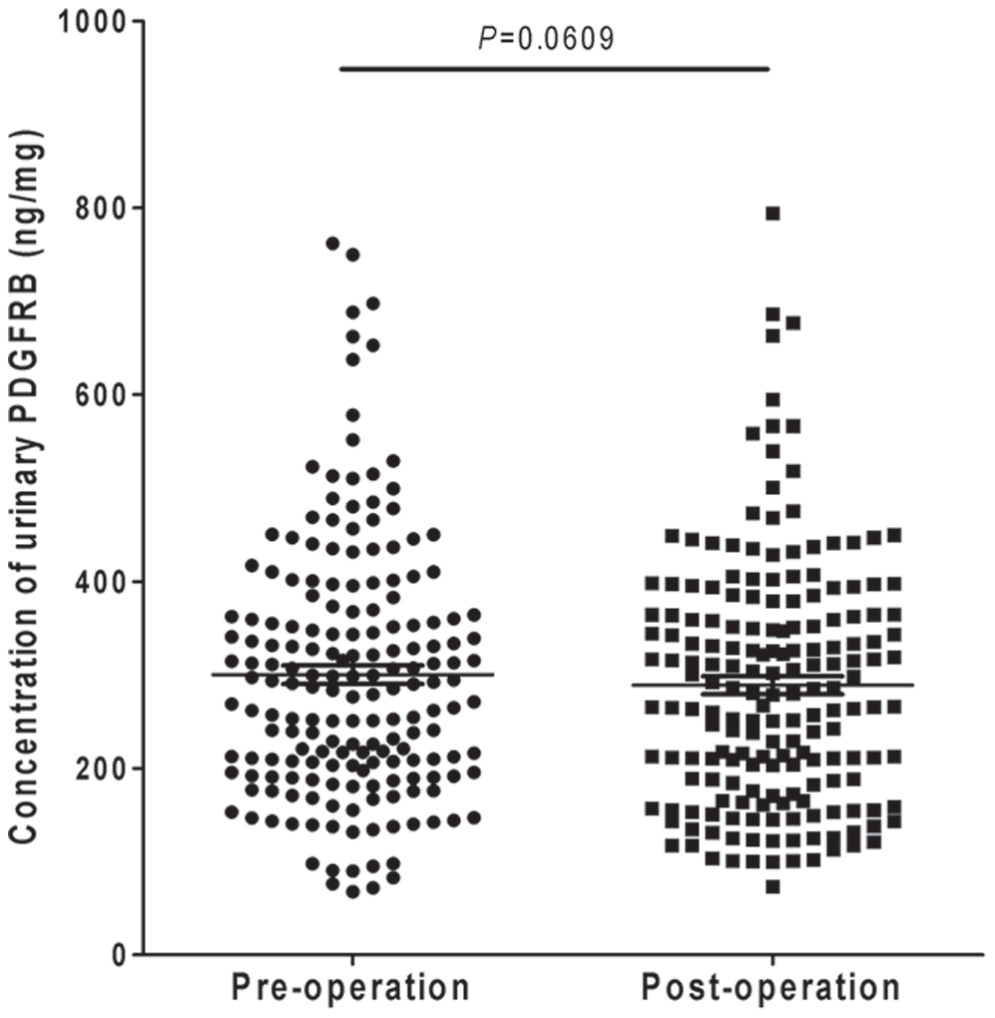
Determination of urinary PDGFRB concentrations in NMIBC patients pre- and post-surgery by ELISA. There was no significant difference between the levels of urinary PDGFRB in NMIBC patients pre-surgery and post-surgery (n = 185) (P = 0.067).

To confirm the preliminary informatics result, urine samples from 185 subjects who were confirmed to have NMIBC (Table 1) were collected before and after surgery. The urinary PDGFRB levels were examined by ELISA. The results showed that the levels of urinary PDGFRB decreased (300.1 ng/mg before surgery vs. 287.23 ng/mg after surgery), but the difference was not significant (P = 0.0607) (Figure 2). This indicated that urinary PDGFRB could not be majorly derived from NMIBC tissue and could not serve as a good early diagnosis biomarker for NMIBC.

### Correlation of the levels of urinary PDGFRB and recurrence of NMIBC

**Figure 3:**
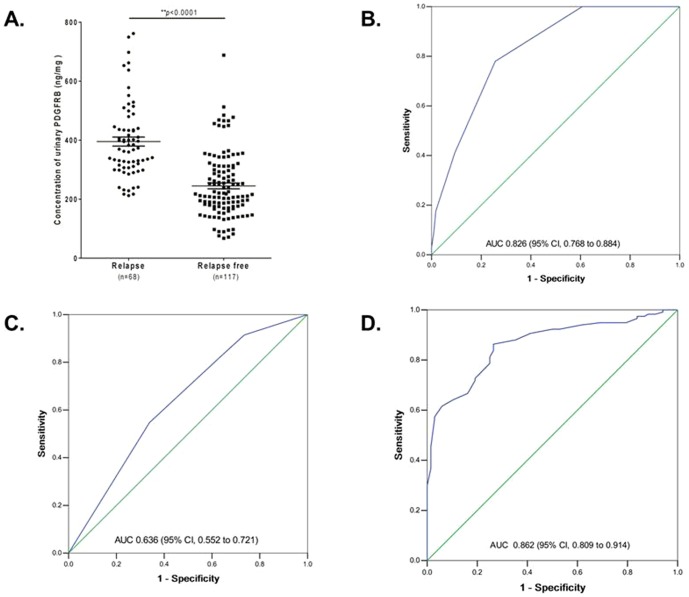
Pre-validation of urinary PDGFRB as a biomarker for predicting the recurrence of NMIBC. (A) Comparison of the level of urinary PDGFRB in relapsed (n = 68) and relapse-free (n = 117) patients with NMIBC. The level of urinary PDGFRB was significantly lower in the patients with recurrence than those without recurrence (P<0.001). (B) The receiver operating characteristics (ROC) curve of urinary PDGFRB. The AUC was 0.826 (95% CI, 0.768 to 0.884). (C) The AUC of age, tumor size, and tumor number combined was 0.636 (95% CI, 0.552 to 0.721). (D) Inclusion of PDGFRB in this model increased the AUC to 0.862 (95% CI, 0.809 to 0.914).

To know whether urinary PDGFRB could be associated with a relapse of NMIBC, a 3-year follow-up study on the 185 NMIBC patients was conducted. The follow-up data showed that among the 185 NMIBC patients, 68 (36.8%) were later found to have recurrence after primary surgery (Table 1). In the relapsed group, the median level of urinary PDGFRB was 395.3 ng/mg (interquartile range 306.65 to 440.01 ng/mg total urinary protein), while it was 245 ng/mg (interquartile range 175.12 to 307.16 ng/mg total urinary protein) for the relapse-free group (Figure 3A, P<0.0001).Indeed, the PDGFRB expression in urine of patient with relapse was confirmed to be significantly increased than that without relapse in 3 years follow-up by Western Blot (Figure S2A in File S1). However, there were no significant differences of the PDGFRB expression on cancer tissue between the patients with relapse and relapse-free in 3 years follow-up by IHC (Figure S2B and S2C in File S1).

A logistic regression model was used to estimate the relationship between urinary PDGFRB and other clinical observations. Significant positive associations were observed between urinary PDGFRB levels and tumor size (P = 0.03) and age (P = 0.019) (Table 4). However, there were no significant associations between urinary PDGFRB and gender, pathological stage, or grade (Table 4). The relationship between urinary PDGFRB and the risk of breast cancer recurrence was further estimated. The result showed that the correlation between urinary PDGFRB levels and the risk of breast cancer recurrence was statistically significant (Table 5, coefficient factor was −1.274, P<0.001). Tumor size (coefficient, −1.193; P = 0.009) and tumor number (coefficient, −0.986; P = 0.022) were also associated with an increased risk of NMIBC recurrence (Table 5). However, other clinical observations such as age, gender, pathological stage, and grade were not significantly associated with the risk of NMIBC recurrence (Table 5).

**Table 4:** Correlation between PDGFRA expression and clinicopathologic factors.

Variables		PDGFRB levels (ng/mg total urinary protein)								*P* value
		0–99	100–199	200–299	300–399	400–499	500–599	600–699	700–799	
	Case	9	37	56	44	25	7	5	2	
Gender										0.205
Female	25	0	6	10	3	5	1	0	0	
Male	160	9	31	46	41	20	6	5	2	
Age (years)										0.030
≤60	81	1	17	25	18	15	5	0	0	
>60	104	8	20	31	26	10	2	5	2	
Tumor size (cm)										0.019
≤3	139	7	34	38	30	22	4	4	0	
>3	46	2	3	18	14	3	3	1	2	
Tumor number										0.235
Unifocal	105	6	25	30	23	12	6	3	0	
Multifocal	80	3	12	26	21	13	1	2	2	
Grade										0.837
I	67	4	13	18	14	12	4	1	1	
II	118	5	24	38	30	13	3	4	1	
T stage										0.717
Ta	74	3	18	24	14	10	3	1	1	
T1	111	6	19	32	30	15	4	4	1	
Recurrence										<0.01
Positive	68	0	0	15	25	16	6	4	2	
Negative	117	9	37	41	19	9	1	1	0	

NOTE: A logistic regression model was used to estimate the odds of PDGFRB adjusted for all of the variables listed in the table.

**Table 5:** Relationship between the biomarker, clinical characteristics and the risk of NMIBC recurrence.

Variable	Coefficient	SE	*P*
Level of urinary PDGFRB	−1.274	.213	<0.001
Gender	−.836	.547	.127
Age	−.548	.402	.172
Tumor size	−1.193	.456	.009
Grade	.042	.476	.930
Pathological stage	.594	.484	.220
Tumor number	−.986	.431	.022

NOTE: A logistic regression model was used to estimate the odds of NMIBC recurrence adjusted for all of the variables listed in the table.

ROC curves were constructed for the clinical factors and PDGFRB. The area under the curve (AUC) of the ROC curve for PDGFRB alone was 0.826 (95% CI, 0.768 to 0.884) (Figure 3B). The AUC of age, tumor size, and tumor number combined was 0.636 (95% CI, 0.552 to 0.721) (Figure 3C). Inclusion of PDGFRB in this model increased the AUC to 0.862 (95% CI, 0.809 to 0.914) (Figure 3D). Using a threshold of 324.12 ng/mg, the sensitivity was 70.6%, while the specificity was 81.2%. When PDGFRB, age, tumor size, and tumor number were combined, the sensitivity increased to 82.7%, and the specificity increased to 88.5%.

## Discussion

The urinary proteome could reflect the urinary system microenvironment, which might play a very important role in urinary system tumor development and progression. Screening the urine for non-invasive biomarkers for bladder cancer is promising. Here, we constructed a cancer-associated PPI network comprised of 15 high-abundance urinary proteins using informatics approach to analyze the urinary proteome of a healthy population (11). Interestingly, of the 15 proteins, 9 (CTNNA3, COL4A6, KLK3, APC, APC2, FGFR2, PDGFRB, EGF, and COL4A2) were identified as being high-abundance proteins in the urinary proteome, but these were not detected or identified as being low abundance proteins in the plasma proteome[17]. This indicates that these proteins were majorly derived from the urinary system cell secretion rather than from plasma and could reflect the characteristic microenvironment of urinary system, which in turn could provide valuable information about the prognosis of a urinary system tumor. Among the 9 proteins, EGF, FGFR2, KLK3, and PDGFRB were associated with urinary system cancer according to the KEGG pathway analysis. Indeed, EGF/EGFR[18], FGFR2 [4], [19], KLK3[20]–[23], and PDGFRB[14] were reported to play important roles in urinary system cancer development and progression. It is well known that the EGF/EGFR pathway plays a critical role in bladder cancer development, progression and recurrence. Although urinary EGFR could serve as a promising prognostic biomarker candidate for bladder cancer, it was identified as a low abundance urinary protein (1 unique peptide and <10 spectrums) and in practice was hard to measure by ELISA (data not shown). Similar to EGFR, FGFR2 and PDGFRB could have the ability to bind EGF (Table 3, STRING database) and transduce the PI3K and MAPK signal pathways[24], [25]. Moreover, PDGFRB rather than FGFR2 could form a heterodimer with EGFR on bladder cancer cells, and this could induce resistance to anti-EGFR therapy for bladder cancer[14]. Together, it is reasonable to assume that PDGFRB is an important receptor for bladder cancer biological progression, and it should serve as a potential biomarker for NMIBC diagnosis and/or prognosis.

The urine is derived from the plasma that is ultrafiltrated by the kidney to eliminate waste products. Unlike serum protein, urinary protein concentration varies with urine dilution. Moreover, the urinary protein from the same individual varies at different times due to the effect of exercise, diet, lifestyle and other factors[10], [26]–[28]. In this study, the variation from urine dilution was eliminated through normalization to total protein, and the variation over time was sufficiently controlled for by using pooled morning urine samples from an individual patient over 3 days before and 3 days after primary surgery (samples were collected once per day). It is known that urinary protein differs by gender and age[26]. Urinary PDGFRB was observed to be significantly associated with age rather than gender in this study (Table 4). In addition, urinary PDGFRB was significantly positively associated with tumor size (Table 4). In 183 NMIBC patients, the level of urinary PDGFRB was increased in patients with a large tumor size (>3 cm; 329.6±22.10 ng/mL, n = 46) compared with those with a small tumor size (≤3 cm; 290.4±11.08 ng/mL, n = 139) (Figure S3 in File S1, P = 0.0907). This suggests that levels of urinary PDGFRB increase with tumor size. Furthermore, there was no significant difference between pre- and post-surgery NMIBC patients (Figure 2, P = 0.0607), which indicates that urinary PDGFRB could not serve as an effective early diagnostic biomarker for NMIBC. In an additional follow-up study, it was observed that the levels of urinary PDGFRB in subjects with relapse were significantly higher than those without recurrence (Fig 3A), and these were also correlated to the risk of 3-year cancer recurrence. The corresponding AUC was 0.826 (Figure 3B; 95% CI, 0.768 to 0.884). Moreover, along with clinical characteristics such as age, tumor size, and tumor number, the AUC increased to 0.862 (Figure 3D; 95% CI, 0.809 to 0.914), which suggests that urinary PDGFRB is a risk factor for NMIBC recurrence and could serve as potential non-invasive biomarker for predicting the recurrence of NMIBC.

### Conclusion

In summary, we for the first time showed that urinary PDGFRB could serve as a non-invasive biomarker for prediction of NMIBC recurrence. Validation by multiple clinical centers is required for further application in clinical settings.

## Supporting Information

File S1 Contains Supporting Figures. Figure S1 Example of network. Figure S2 Validation of relapse. Figure S3 Determination of urinary PDGFRB. **Figure S1. The PPI network was analyzed by Cytoscape software with the ClueGO+Cluepedia plug-in.** The enriched KEGG terms were shown. **Figure S2. Validation of PDGFRB expression in urine and on tumor tissues of NMIBC patients with relapse and relapse-free.** The expression of PDGFRB in urine of NMIBC patients with relapse and relapse-free was analyzed by Western Blotting (A). The expression of PDGFRB on tumor tissue of NMIBC patients with relapse (B) and relapse-free (C) was analyzed by IHC. A total of 50 cancer tissues from 27 relapsed and 23 relapse-free patients were evaluated through immunohistochemistry. There were no significant differences of PDGFR expression on cancer tissues between relapsed and relapse-free groups. The representative 6 samples from relapsed and relapse-free patients were showed in 3A and 3B, respectively. **Figure S3. Determination of urinary PDGFRB concentrations in NMIBC patients with a large and small tumor size by ELISA.** In 183 NMIBC patients, the level of urinary PDGFRB was increased in patients with a large tumor size (>3 cm) compared with those with a small tumor size (≤3 cm)(P = 0.0906).
(PDF)

File S2 Supplemental Data.
(XLS)
