# Supplementary figures and images for: Platelet-Derived Growth Factor Receptor Beta: A Novel Urinary Biomarker for Recurrence of Non-Muscle-Invasive Bladder Cancer
Source: PLoS One. 2014 May 6;9(5):e96671. doi: 10.1371/journal.pone.0096671 (PMC4011858; doi:10.1371/journal.pone.0096671)

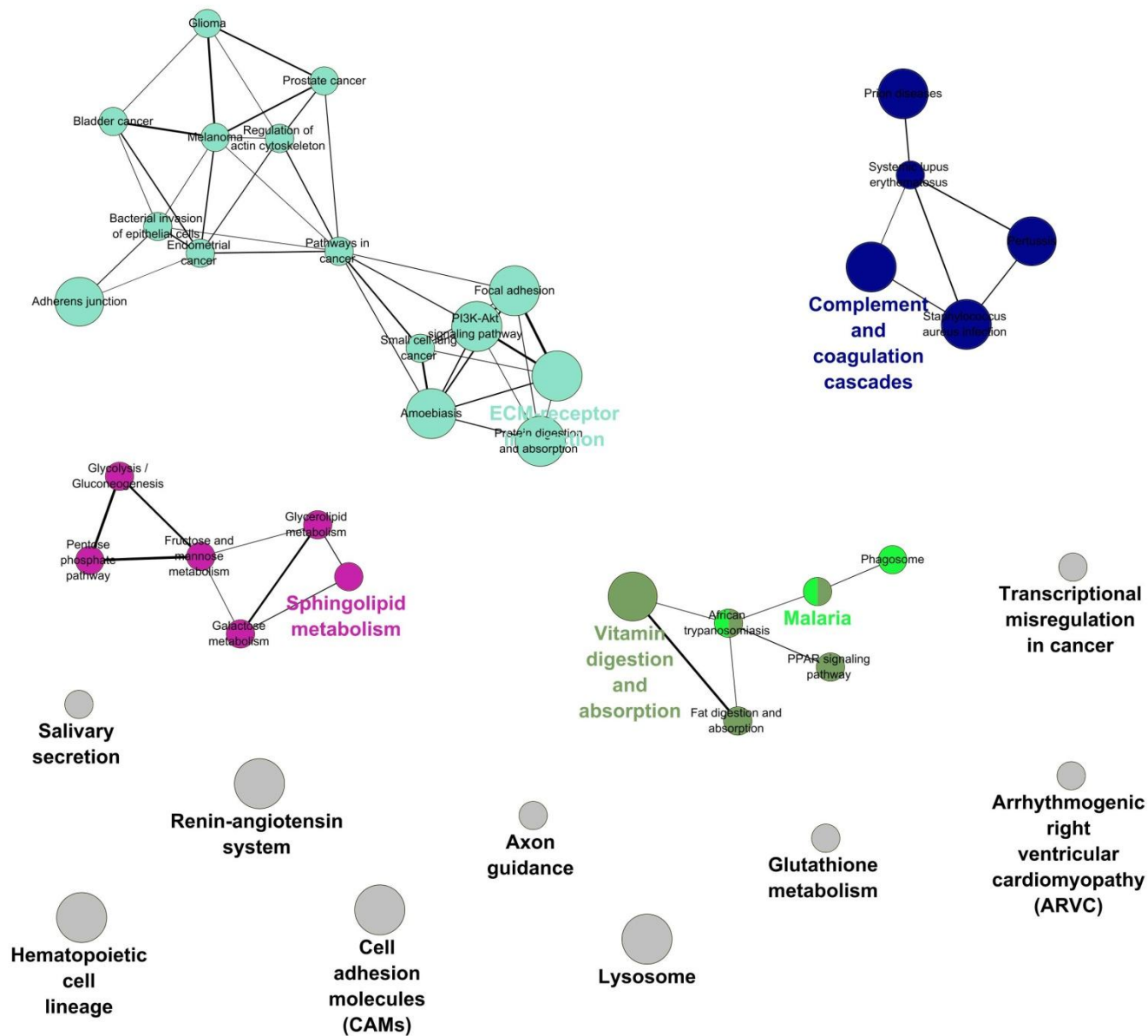

Figure S1

**A.**

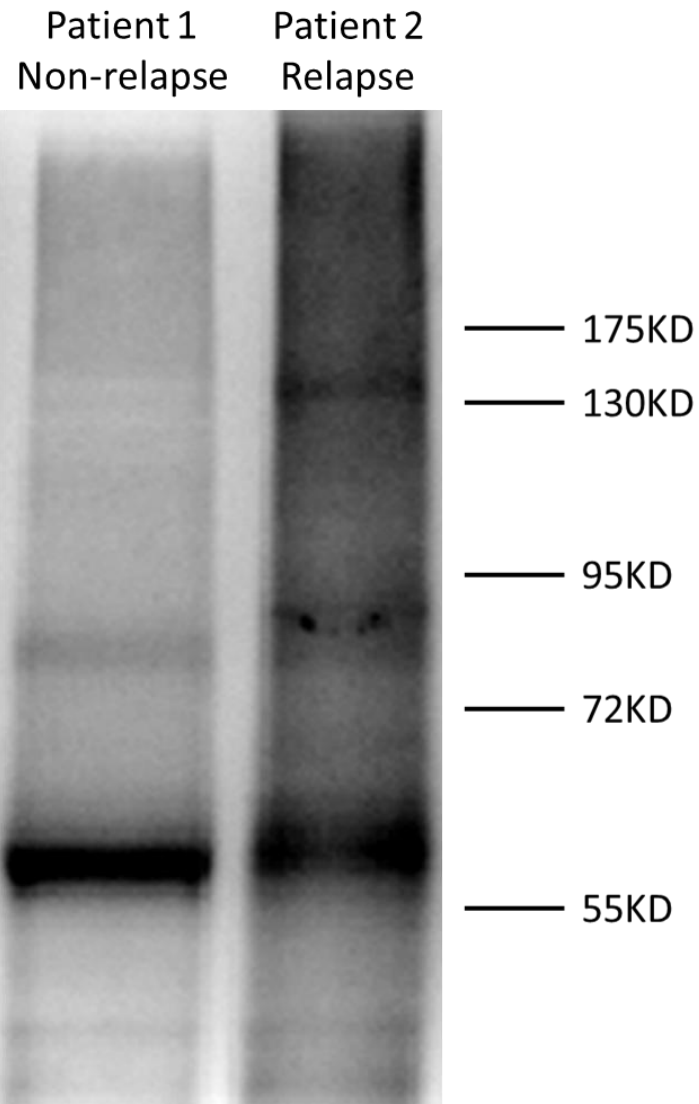

Figure S2

**B.**

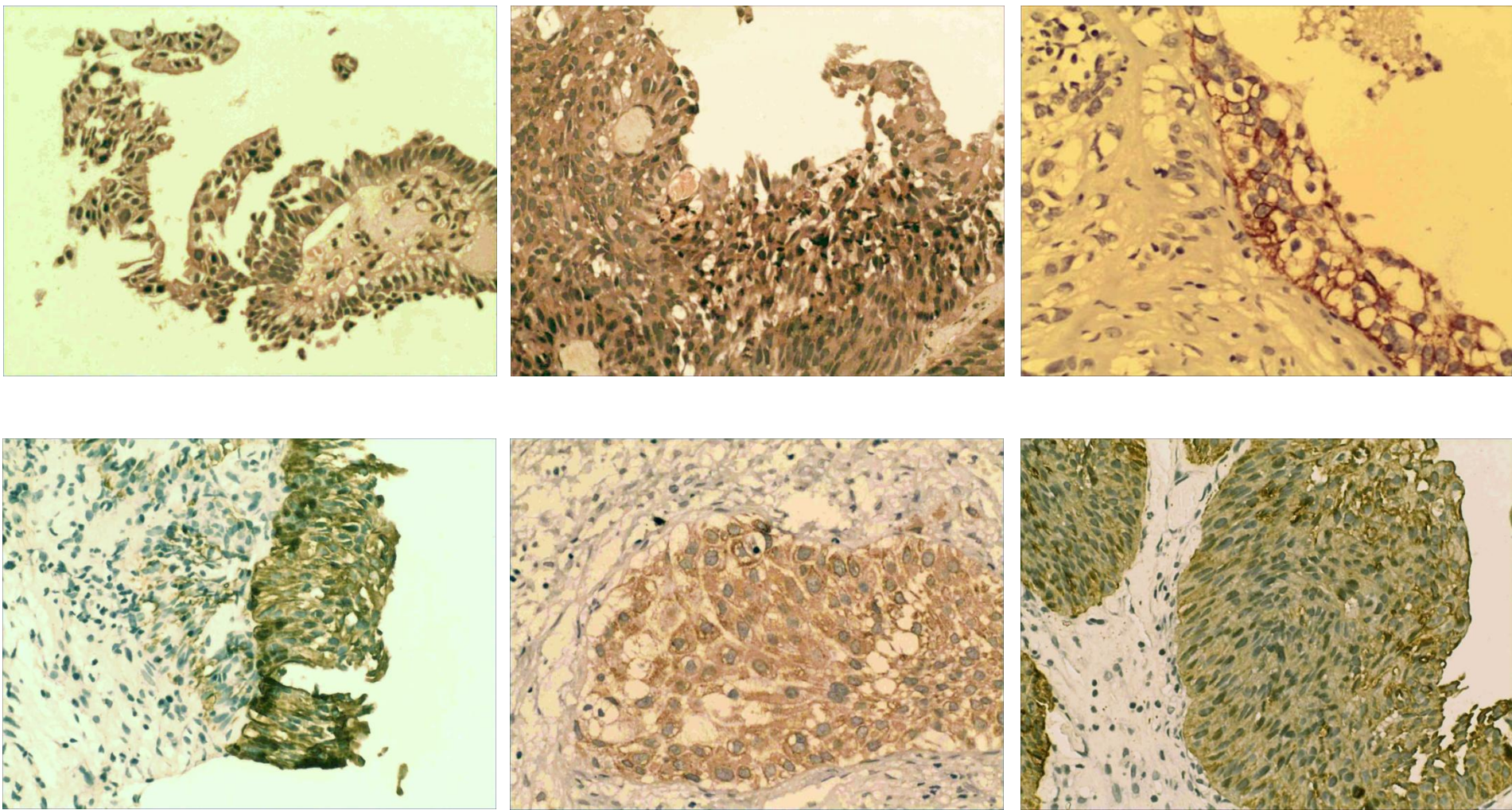

Figure S2

C.

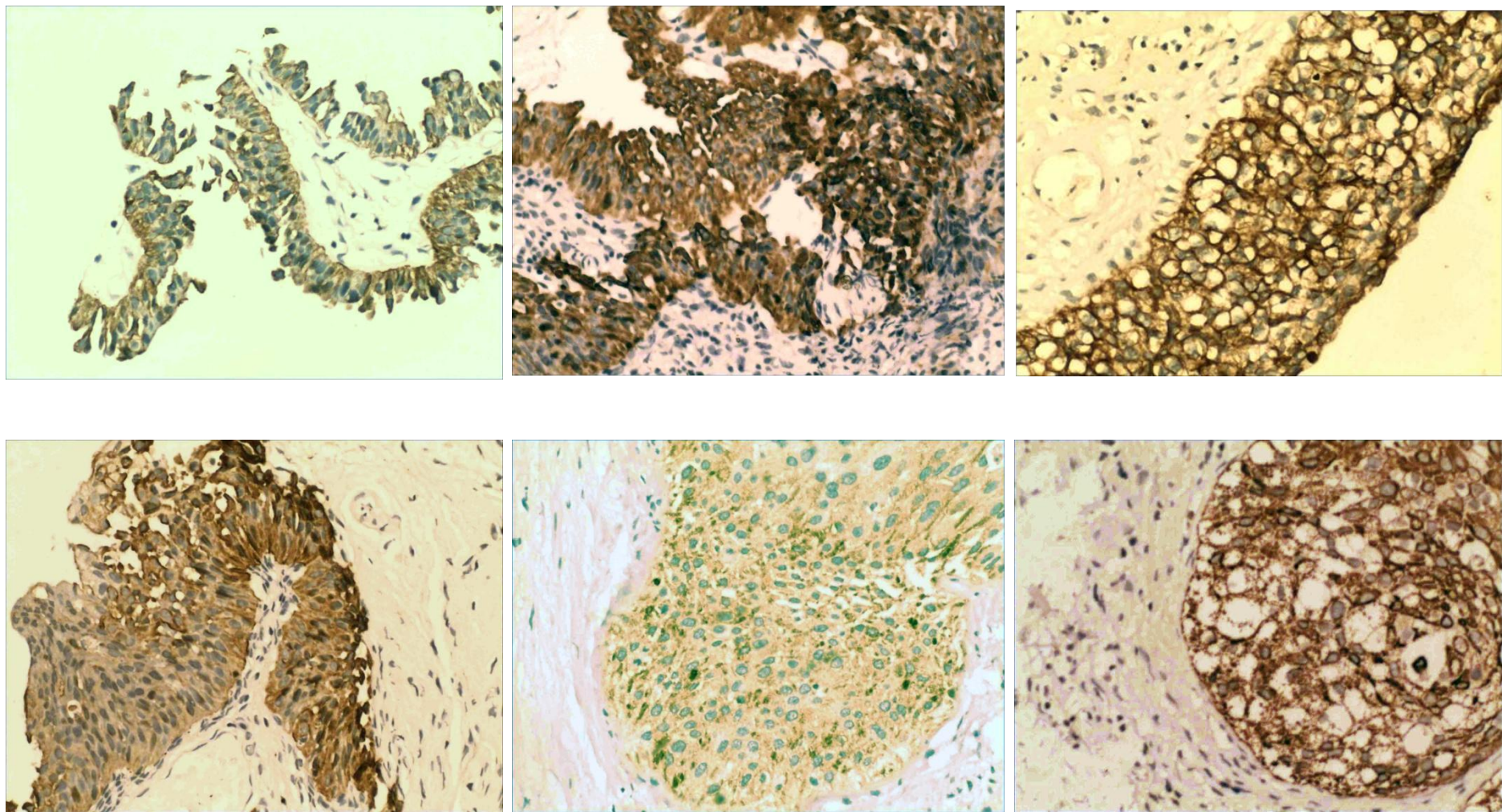

Figure S2

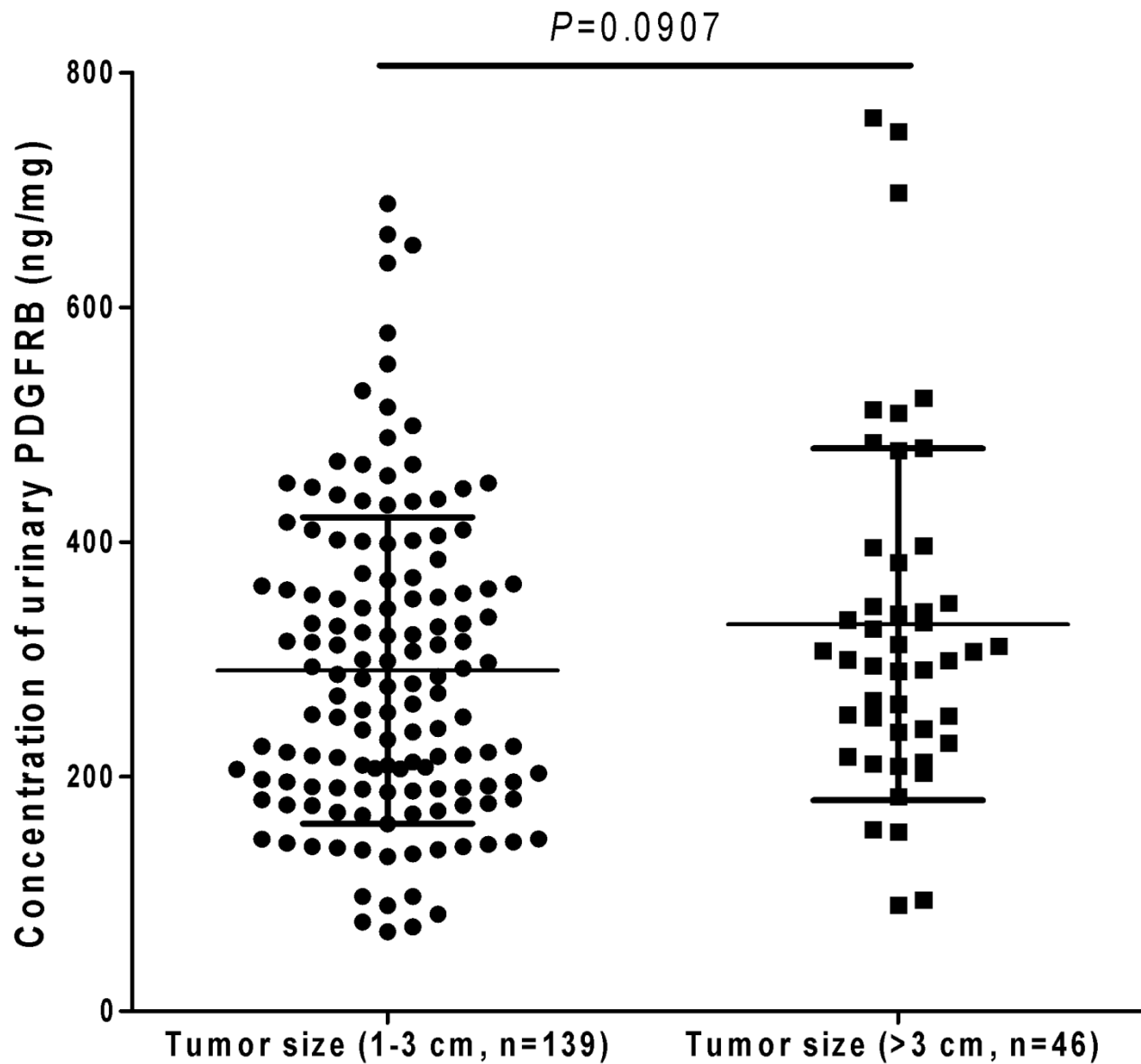

Figure S3

Supplement: File S1 — Contains Supporting Figures. Figure S1 Example of network. Figure S2 Validation of relapse. Figure S3 Determination of urinary PDGFRB. Figure S1. The PPI network was analyzed by Cytoscape software with the ClueGO+Cluepedia plug-in. The enriched KEGG terms were shown. Figure S2. Validation of PDGFRB expression in urine and on tumor tissues of NMIBC patients with relapse and relapse-free. The expression of PDGFRB in urine of NMIBC patients with relapse and relapse-free was analyzed by Western Blotting (A). The expression of PDGFRB on tumor tissue of NMIBC patients with relapse (B) and relapse-free (C) was analyzed by IHC. A total of 50 cancer tissues from 27 relapsed and 23 relapse-free patients were evaluated through immunohistochemistry. There were no significant differences of PDGFR expression on cancer tissues between relapsed and relapse-free groups. The representative 6 samples from relapsed and relapse-free patients were showed in 3A and 3B, respectively. Figure S3. Determination of urinary PDGFRB concentrations in NMIBC patients with a large and small tumor size by ELISA. In 183 NMIBC patients, the level of urinary PDGFRB was increased in patients with a large tumor size (>3 cm) compared with those with a small tumor size (≤3 cm)(P = 0.0906). (PDF) [file pone.0096671.s001.pdf]
